# Characterizing hepatocellular carcinoma stem markers and their corresponding susceptibility to NK-cell based immunotherapy

**DOI:** 10.3389/fimmu.2023.1284669

**Published:** 2023-10-26

**Authors:** Jason Chiang, Po-Chun Chen, Janet Pham, Cat-Quynh Nguyen, Kawaljit Kaur, Steven S. Raman, Anahid Jewett

**Affiliations:** ^1^ Department of Radiology, Ronald Reagan UCLA Medical Center, Los Angeles, CA, United States; ^2^ The Jonsson Comprehensive Cancer Center, UCLA School of Dentistry and Medicine, Los Angeles, CA, United States; ^3^ Division of Oral Biology and Medicine, The Jane and Jerry Weintraub Center for Reconstructive Biotechnology, UCLA School of Dentistry, Los Angeles, CA, United States

**Keywords:** hepatocellular carcinoma, NK cells therapy, stem-like cancer cells, immunotherapy, hepatobiliary system

## Abstract

**Introduction:**

Hepatocellular carcinoma (HCC) is the most common primary liver cancer and is the fourth-leading cause of all cancer-related deaths around the world. Liver transplantation, surgery, and local ablation are curative therapies for early-stage HCC. However, post-treatment outcomes can vary based on histopathologic stage. Poorly-differentiated HCC are associated with higher rates of tumor progression and lower overall survival compared to well-differentiated HCC after therapy. In this study, we aimed to characterize the cancer stem cell (CSC) profile of histopathologically-proven well and poorly-differentiated HCCs in an *in-vitro* environment. We characterized the stem-like profile of each type of HCC based on their surface markers and susceptibility to NK cell-mediated cytotoxicity.

**Methods:**

Flow cytometry was used to quantify differential expression of MHC-class I, CD54, and CD44 between well- and poorly-differentiated HCCs. Primary untreated NK cells, IL-2 stimulated primary NK cells, and supercharged (sNK) cell-mediated cytotoxicity was assessed against well- and poorly-differentiated HCCs. IFN-γ supernatant from each respective NK cell experimental arm was also used to induce differentiation of HCCs. Finally, we characterized the temporal NK effector cell cytotoxicity using real-time quantitative analysis of imaging and impedance (eSight study).

**Results:**

Poorly-differentiated HCCs demonstrated low surface expression of MHC-class I and CD54, and high expression of CD44. Treatment of NK cells secreted IFN-γ or IFN-γ cytokine induced differentiation in HCCs. Poorly-differentiated HCCs in comparison to well-differentiated HCC were more susceptible to NK cell-mediated cytotoxicity in primary NK cells, IL-2 stimulated primary NK cells, and sNK cells. sNK cells induced significantly higher cytotoxicity against well-differentiated HCCs in comparison to untreated or IL-2-stimulated primary NK cells. These findings were recapitulated with real-time quantitative imaging analysis.

**Conclusions:**

Poorly-differentiated HCCs were found to have surface marker patterns of CSCs, making them highly susceptible to NK cell-based immunotherapy. NK-cell based therapy can potentially be leveraged as a neoadjuvant or adjuvant therapy in poorly-differentiated HCCs. Supercharged NK cells, which can be rapidly expanded to therapeutic levels, are uniquely capable of lysing both poorly- and well-differentiated HCCs. This finding suggests that sNK cells not only exhibit enhanced features against NK cells’ targets but also are capable of activating T cells to induce cytotoxicity against well-differentiated HCCs with high expression of MHC class I.

## Introduction

Hepatocellular carcinoma (HCC) is the most common form of primary liver cancer worldwide and continues to rise in incidence from increasing rates of non-alcoholic steatohepatitis and hepatitis B and C (1). The Barcelona Clinic Liver Cancer (BCLC) staging criteria provides a framework for treating HCC based on a combination of disease stage, liver function, and the patient’s performance status (2). For early-stage HCC, liver transplantation remains the only curative option that simultaneously treats both the liver tumor and the underlying cirrhosis. However, liver transplant outcomes are not only influenced by underlying patient comorbidities and surgical technique, but also the grade of their respective histopathologic HCC disease. Multiple prior studies have shown that liver transplant recipients with poorly-differentiated HCCs in their native liver had significantly lower overall disease-free and overall survival rates (3, 4). This trend has also been seen in patients undergoing surgical resection and percutaneous thermal ablation, where more poorly differentiated HCCs were similarly associated with suboptimal outcomes (5, 6). As a result, there has been increasing interest in identifying features of tumor differentiation prior to treatment to help optimize post-treatment outcomes.

The stage of tumor differentiation is primarily diagnosed on surgical resection or liver explant histopathology. Well-differentiated tumors have differentiation markers that are similar to those seen in normal tissue of the background liver, whereas poorly differentiated markers lack these respective “self” markers. The downregulation or upregulation of specific differentiation markers can be grouped together and associated with cancer stem cell (CSC) traits (7). These CSC traits refer to specific stem-cell-like features of self-renewal and differentiation, behaviors that play a key role in tumor metastasis and post-treatment recurrence. Given their association with cancer aggressiveness, identifying these markers is an area of active investigation, with low MHC-class I and high CD44 being noted as potential markers of liver CSCs (8–11). Normally, the immune system can control the CSC population by identifying and eliminating tumor cells with CSC differentiation markers. However, altered tumor microenvironments can dampen the immune response and aid in the growth of CSCs and subsequent cancer propagation. This is particularly true with HCC, of which greater than 90% grow under conditions of chronic inflammation (8). Consequently, while immune surveillance can identify nascent transformed CSCs via the innate and adaptive immune response, its efficacy may be compromised in the setting of chronic inflammation. The continuous reciprocal communication between liver CSCs and infiltrating immune cell population ultimately facilitates immune evasion of the tumor.

An important link between markers of stemness in tumor biology is that they have been hypothesized to be preferentially susceptible to natural killer (NK) cell-based therapy (12–15). NK cells are an essential component of innate immunity that do not require antigen priming or MHC-mediated interactions with target tumor cells. NK cell activity is modulated by germline-encoded surface receptors that bind to multiple activating or inhibitory ligands and co-receptors. Because of their MHC-independence, NK cells have become a major vehicle for novel cell-based immunotherapies. With over half of the lymphocytes in the human liver comprising of NK cells, it has been noted that peripheral and intrahepatic NK cell population can serve as a biomarker to predict HCC patient clinical outcomes (16). Patients with decreased expression of MHC-class I chain-related protein A (MICA) in their HCC tissue had shorter disease free and overall survival compared to patients with HCC samples with preserved MHC-class I expression (17).

NK-cell based immunotherapy has been hypothesized to have strong potential in treating solid tumors, but there are still challenges in its successful translation to human trials. NK cells are limited in number within peripheral blood and require functionalization in an *in-vitro* environment to persist in the tumor microenvironment (18, 19). Furthermore, NK cells have been difficult to expand to levels needed for cell-based therapies (20). A novel expansion methodology using a combination of osteoclasts (OCs) and probiotic bacteria have been leveraged to generate highly activated “supercharged” NK (sNK) cells (21). These sNK cells have demonstrated strong anti-tumor activity both *in-vitro* and *in-vivo* against poorly-differentiated CSCs of solid tumors such as oral squamous cell carcinoma, pancreatic cancer, and melanoma (15, 22–24) (manuscript in prep.).

Poorly differentiated HCCs have been known to be aggressive and difficult to treat with conventional surgery or ablation. NK-cell based immunotherapy of HCC presents a unique opportunity for therapy in this underserved population, especially given the fact that NK cells can attack poorly differentiated cells with downregulated MCH-class I molecules. The goal of this study was to evaluate the CSC profile of human well- and poorly-differentiated HCC cell lines in an *in-vitro* environment. Characterization of the stem-like profile of each type of HCC was validated based on their surface markers and their susceptivity to NK cell-mediated cytotoxicity using primary untreated NK cells, primary IL-2 stimulated NK cells, and sNK cells.

## Materials and methods

### Cell lines, reagents, and antibodies

RPMI 1640 supplemented with 10% fetal bovine serum (FBS) (Gemini Bio-Products, CA), 2% of 100X antibiotic/antimycotic solution (Cytiva, MA), 1% of 100mM sodium pyruvate (Gibco, CA), and 1% of 100X MEM non-essential amino acids (Gibco, CA) was used to culture NK cells. Oral squamous carcinoma stem cells (OSCSCs) and oral squamous cell carcinoma (OSCCs), which served as cancer stem and non-stem control groups, respectively, were extracted from patients with tongue tumors at the University of California, Los Angeles (UCLA) (15, 24–26). The OSCSCs and OSCCs were maintained in RPMI 1640 medium (Gibco, ThermoFisher, CA) supplemented with 10% fetal bovine serum (FBS) (Gemini Bio-Products, CA), 2% of 100X antibiotic/antimycotic solution (Cytiva, MA), 1.4% of 100mM sodium pyruvate (Gibco, CA), and 1.4% of 100X MEM non-essential amino acids (Gibco, CA). The cell lines HepG2 and SNU-423 were procured from the American Type Culture Collection (ATCC) and were cultured in DMEM and RPMI 1640 medium (Gibco, ThermoFisher, CA) respectively and both were supplemented with 10% fetal bovine serum (FBS) (Gemini Bio-Products, CA) and 2% of 100X antibiotic/antimycotic solution (Cytiva, MA). Recombinant IL-2 was obtained from Hoffman La Roche (NJ, USA). The Lymphoprep and NK enrichment kit were acquired from Stemcell Technologies (Cambridge, MA). Antibodies used for flow cytometry were: CD44, CD54, MHC- class I, CD3, CD16, CD56 and CD45; all of which were purchased from Biolegend (San Diego, CA). Chromium-51 (^51^Cr) was purchased from PerkinElmer (Waltham, MA). Heated, supernatant-treated, and wildtype OSCSCs, OSCCs, HepG2, and SNU-423 cells were washed with 1X PBS once after being detached from the culture dish with 0.25% Trypsin-EDTA (Gibco, ThermoFisher, CA).

### Surface marker analysis using flow cytometry

For surface marker analysis, all cells were washed twice using ice-cold PBS+1%BSA. Predetermined optimal concentrations of specific human monoclonal antibodies were added to 1 x 10^4^ cells in 100 µl of cold PBS+1%BSA, and were incubated on ice for 30 min in a dark room. Thereafter, cells were washed in cold PBS+1%BSA and brought to 200 µl with PBS+1%BSA. Flow cytometry data was analyzed using an Attune NxT flow cytometer (ThermoFisher) with Attune NxT software v5.2.0. Data were analyzed using FlowJo (v10.7) software.

### Purification of NK cells from human peripheral blood

Written informed consent was obtained from healthy blood donors, approved by the UCLA-IRB (IRB#11-000781). Peripheral blood was separated by differential centrifugation (Lymphoprep Density Gradient Medium, Stemcell Technologies, Vancouver). The cloudy white buffy coat containing peripheral blood mononuclear cells (PBMCs) was collected, washed, and resuspended in RPMI 1640 supplemented with 10% FBS. NK cells were isolated from PBMCs using the Human NK Cell Enrichment Kit (Stemcell Technologies, Vancouver) by negative selection. The isolated NK cells were then stained with anti-CD45, anti-CD16, anti-CD56, and anti-CD3 antibodies to measure cell purity by flow cytometry analysis. Purified NK cells were cultured in RPMI 1640 medium supplemented with 10% FBS (Gemini BioProducts, CA), 2% antibiotics/antifungals (Cytiva, MA), 1% sodium pyruvate (Gibco, CA), and 1% non-essential amino acid MEM (Invitrogen, Life Technologies, CA). All NK cells used in the experiments had a purity above 90%.

### Generation of supercharged NK (sNK) cells

The method to generate sNK cells has been described in-depth in a previous paper (21). Briefly, purified human NK cells were activated with rh-IL-2 (5000 IU/ml) and anti-CD16 mAbs (3 ug/ml) for 18-20 hours, and then co-cultured with osteoclasts (OCs) and sonicated AJ2 (combination of 8 different strains of gram-positive probiotic bacteria) in an NK: OCs: sAJ2 mixture in a 2:1:4 ratio (24, 27). The medium was renewed with RPMI supplemented with rhIL-2 (2000 IU/mL) every three days.

### Primary NK and sNK cells’ supernatant collection and tumor cell differentiation

Primary NK cells were activated using either rh-IL-2 (5000 U/mL) alone or rh-IL-2 (5000 U/mL) with anti-CD16 mAbs (3 μg/mL) for 48 hours prior to supernatant collection. For sNK cells, the supernatant was harvested on day 14 of culture to measure the levels of IFN-γ using ELISA. The volume of supernatant used was based on a pre-determined amount of IFN-γ needed previously required to induce differentiation of SNU-423 and HepG2. On day 0, 3 x 10^5^ tumor cells were cultured in 2 mL of media within each well of a 6-well plate. The tumor cells were treated with either the supernatant of IL-2-treated primary NK, IL-2+anti-CD16 mAbs-treated primary NK, sNK, or recombinant hu-IFN-γ, using concentrations of either 100 pg/mL or 10 ng/mL IFN-γ for two days. On day 6, the tumor cells were rinsed with 1X PBS, detached, and were used for surface marker analysis using flow cytometry.

### NK cell-mediated cytotoxicity against HCC cell lines using chromium release assay

The ^51^Cr release cytotoxicity assay was performed as previously described (27). Briefly, a serial 2-fold dilution was created to make an E:T ratio of 5:1 starting with 5 x 10^4^ NK effector cells. ^51^Cr–labeled OSCC, OSCSCs, HepG2, and SNU-423 cells were incubated together for four hours. Afterwards, the supernatants were harvested from each sample, and the released radioactivity was counted for each using the gamma counter. The percentage specific cytotoxicity was calculated as follows:


$$\text{\%cytotoxicity}=\frac{\text{Experimental cpm}-\text{spontaneous cpm}}{\text{Total cpm}-\text{spontaneous cpm}}$$

LU 30/10^6^ was calculated by using the inverse of the number of effector NK cells needed to lyse 30% of tumors ×100.

### Monitoring *in-vitro* NK cell-mediated killing of cells

NK cell-mediated killing was measured via the xCELLigence RTCA eSight platform. HCC cell lines were seeded at a density of 1x10^4^ cells/100 μL using the same growth medium and left to incubate for 24 hours to facilitate attachment. Primary NK cells were isolated from healthy donors as described above. HCC cell lines and OSCSCs were treated with primary untreated NK cells, rh-IL-2 (5000 U/mL) alone, or rh-IL-2 (5000 U/mL) with anti-CD16 mAbs (3 μg/mL) stimulated primary NK cells, and sNK cells. Incubation with each type of NK-cell therapy was monitored over the course of the experiment via quantitative analysis of imaging and impedance. The impedance-based xCELLigence technology utilizes proprietary microplates (E-Plates View 96) embedded with gold biosensors at the bottom of each well, which serve to non-invasively quantify cell behavior. Over the course of an experiment, the biosensors quantitatively measured cell metrics such as proliferation, adhesion strength, changes in morphology, migration, and differentiation. For impedance and cell viability measurements, the cell index and percent cytolysis were calculated every 15 minutes and images were acquired every hour over the course of 85 hours. Percent cytolysis was defined as [1-Normalized cell index (treatment group)/Normalized cell index (control group)]x100. Cell indices less than zero were interpreted as total cell death from effector cell cytotoxicity.

### Statistical analyses

Differences among treatment groups were analyzed using Student’s t-test for normally distributed variables. A p-value of<0.05 was considered statistically significant. Statistical analysis was performed using GraphPad Prism v.9 (GraphPad Software Inc). The following symbols represents the level of statistical significance within each analysis: **** (p-value<0.0001); *** (p-value 0.0001-0.001), **(p-value 0.001-0.01), *(p-value 0.01-0.05).

## Results

### HCC immunlogic stem profile corresponded to the grade of histopathologic differentiation based on cell growth and surface markers

**Figure 1:**
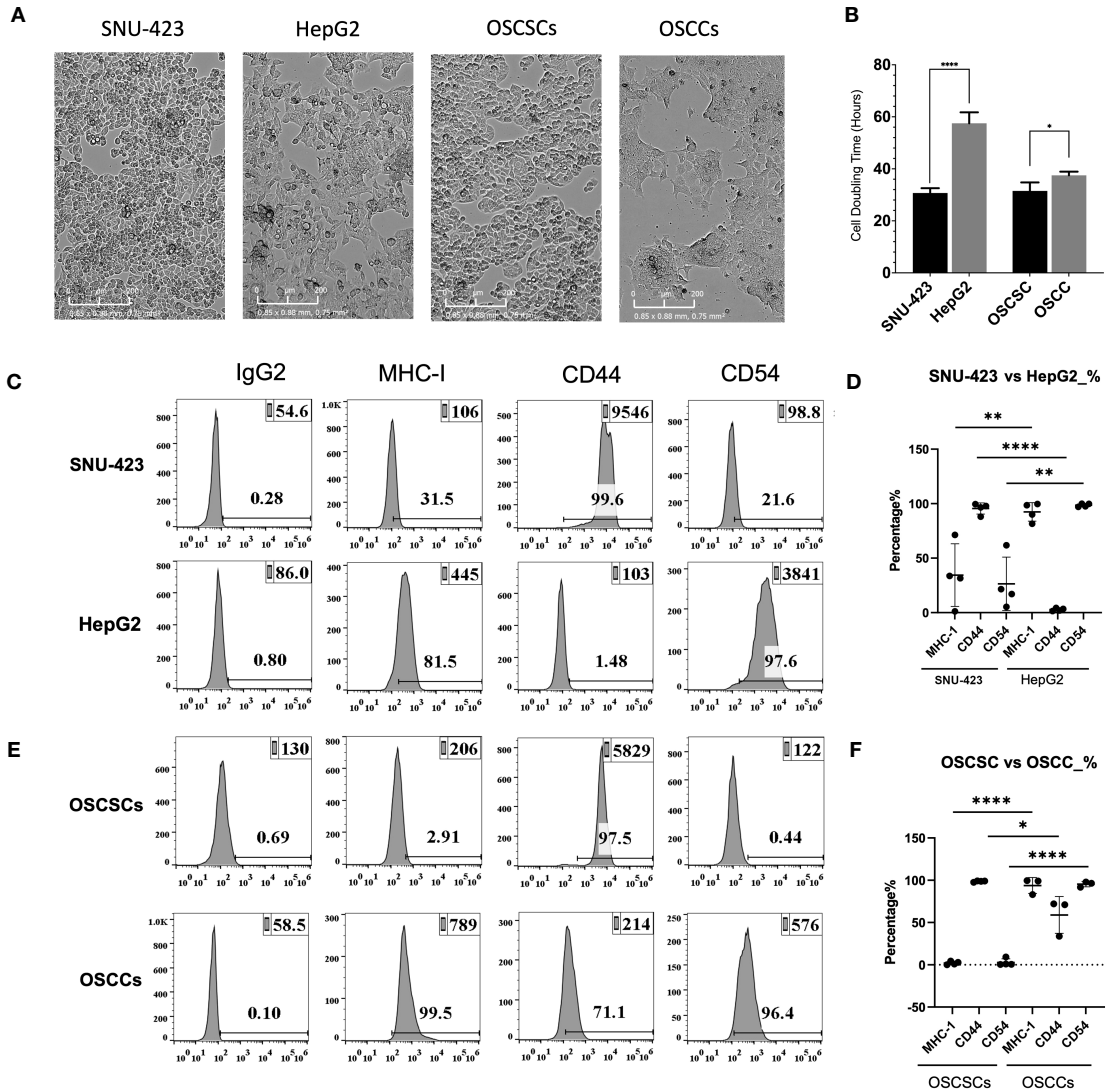
HCC immunologic stem profile correlates with the grade of histopathologic differentiation. **(A)** Microscopy of poorly-differentiated HCC (SNU-423) and well-differentiated HCC (HepG2), seen in the left two panels. These poorly- and well-differentiated HCC cell lines were compared against previously established poorly-differentiated oral squamous stem-like cells and well-differentiated oral squamous cell carcinoma, respectively. **(B)** Poorly-differentiated HCC (SNU-423) had a significantly shorter doubling time compared to well-differentiated HCC (HepG2) (30.7 ± 2.0 vs 57.5 ± 5.9 hours; p<0.0001). OSCSCs similarly had a shorter doubling time compared to OSCSC (31.5 ±3.9 vs 37.5 ±1.7 hours; p<0.01). **(C)** Poorly-differentiated HCCs (SNU-423) exhibited immunologic stem-profiles, with lower levels of MHC-I and CD54, as well as higher levels of CD44 compared to well-differentiated HCC (HepG2). **(D)** When subtracting out the IgG control florescence, SNU-423 had significantly lower fluorescence in MHC-I (34.4 ± 28.7 vs 92.5 ± 8.5%; p=0.008) and CD54 (26.5 ± 24.6 vs 98.6 ±1.5%; p=0.001) compared to HepG2. SNU-423 also demonstrated significantly higher expression of stem cell marker CD44 (95.5 ± 5.1 vs 2.8 ± 1.3%; p<0.0001). **(E)** This trend was seen in the oral squamous cell carcinoma stem cells (OSCSC) and oral squamous cell carcinoma (OSCC), where OSCSC had lower expression of MHC-I and CD54, and higher expression of CD44. **(F)** OSCSC showed significantly lower fluorescence contribution of MHC-I and CD54, and higher contribution of CD44. These trends confirm that poorly-differentiated HCC have similar immunologic stem-profiles as previously characterized stem-like tumors. The following symbols represents the level of statistical significance within each analysis: **** (p-value<0.0001); *** (p-value 0.0001-0.001), **(p-value 0.001-0.01), *(p-value 0.01-0.05).

Prior studies have demonstrated differences between CSCs and differentiated tumors based on surface markers (MHC-class I, CD54 and CD44) and their growth rate *in-vitro* (15, 22, 24, 25, 28–30). In this study, the stem-like profile of poorly-differentiated HCCs and oral squamous stem-like carcinoma (OSCSCs) were compared against well-differentiated HCCs and oral squamous cell carcinoma (OSCCs), respectively (**Figure 1A**). Poorly-differentiated HCCs (SNU-423) had significantly shorter doubling time compared to well-differentiated HCCs (HepG2), a trend that is also seen when comparing OSCSCs against OSCCs (**Figure 1B** **).** Poorly-differentiated HCCs (SNU-423) in comparison to well-differentiated HCCs (HepG2) exhibited lower MHC-class I and CD54, and higher CD44 surface expression levels (**Figures 1C, D**). OSCSCs, when compared to OSCCs, had exhibited a similar trend, with lower MHC-class I and CD54, and higher CD44 surface expression levels (**Figures 1E, F**).

### Supercharged NK cells expanded at higher rates and mediated higher levels of cytotoxicity against both CSCs and well-differentiated HCC tumors in comparison to primary untreated and IL-2 activated NK cells

**Figure 2:**
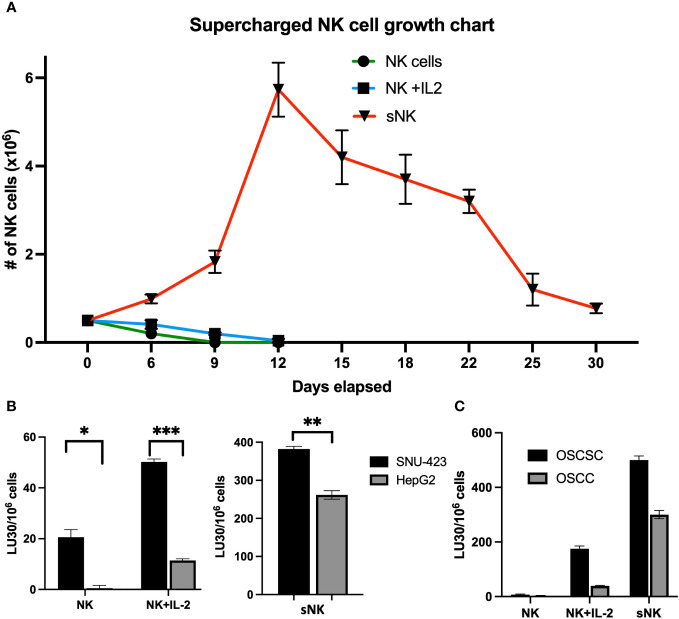
NK cell cytotoxicity against well-differentiated and poorly-differentiated HCC. **(A)** Supercharged NK (sNK) cells are a rapidly-expanded type of NK cell that is characterized by higher levels of growth and functional activation. In this graph, sNK cells have a cell population that is 140x higher compared to IL-2 activated NK cells (5.73 x 10^6^ sNK cells vs 4.3 x 10^4^ IL-2 activated NK cells at day 12) (Kaur et al. Front. Immunol. 2023; CC BY 4.0). Note the rapid upward growth rate of sNK cells compared to both peripheral NK and IL-2 stimulated NK cells, of which both rapidly decline after plating. **(B)** Primary NK cells were confirmed to have higher levels of activity against poorly-differentiated HCC (SNU-423) compared to well-differentiate HCC (HepG2) (20.6 ± 3.0 vs 0.5 ± 1.1 LU30/10^6^ cells; p=0.013) via chromium assay. This trend was amplified in the IL-2 activated NK cells (NK+IL-2) where there was preferentially higher levels of cytotoxicity in SNU-423 compared to HepG2 (50.2 ± 1.1 vs 11.4± 0.7 LU30/10^6^ cells; p<0.001). As expected, IL-2 activated NK cells had increased activity against both HepG2 and SNU-423 compared to peripheral NK cells alone (left) Supercharged NK cells exhibit higher activity levels against both HepG2 and SNU-423, with preferential activity toward SNU-423 (382.6 ± 6.7 vs 261.7 ± 11.1 LU30/10^6^ cells; p=0.006). Note that sNK cell activity is several times higher when compared to IL-2 stimulated NK cells with SNU-423 (382.6 ± 6.7 vs 50.2 ± 1.1 LU30/10^6^ cells; p<0.001) and HepG2 (261.7 ± 11.1 vs 11.4 ±0.7 LU30/10^6^ cells; p<0.001) (right). **(C)** The escalating cytotoxicity from peripheral NK to IL-stimulated NK and supercharged NK cells is recapitulated in the oral squamous cell carcinoma (OSCC) and oral squamous cell stem cell (OSCSC) lines, with preferential activity against OSCSC due to stem features. The following symbols represents the level of statistical significance within each analysis: *** (p-value 0.0001-0.001), **(p-value 0.001-0.01), *(p-value 0.01-0.05).

CSCs have been shown to be more susceptible to NK cell-mediated cytotoxicity compared to their well-differentiated counterparts (15, 22, 24, 25, 28–30). However, primary NK cells are difficult to expand *in-vitro* to levels required for cell-based therapy. Supercharged NK (sNK) cells exhibit uniquely high expansion rates and potent anti-tumor function compared to primary NK cells (21, 31). Cell expansion rates were compared between untreated primary or IL-2 stimulated primary NK cells, and sNK. sNK cells were noted to have the highest cell expansion rate with a peak sNK cell population that was greater than 140-fold higher compared to IL-2 activated NK cells (**Figure 2A**). In terms of the number of expanded cells, based on fold expansion from a prior study, sNK cells were able to be expanded to >1 million-fold after 31 days (21). No substantial apoptosis was observed during the study, with 4% PI-positive sNK cells after 36 days. However, sNK cells required stimulation with osteoclasts at day 36 and 60 to continue its rate of expansion. sNK cells also demonstrated a different phenotype compared to IL-2 stimulated NK cells, with higher expression of activating receptors Nkp30, Nkp44, Nkp46, KIR3, CD94 and NKG2D. Over the course of their expansion sNK cells showed an initial decrease in proportion of CD3^-^CD56^+^ cells but eventually returned to baseline by day 21 (21, 32).

As expected, both untreated and IL-2 activated primary NK cells induced higher cytotoxicity against poorly-differentiated HCCs (SNU-423) compared to the well-differentiated HCC (HepG2) (**Figure 2B**). sNK cells also induced significantly higher levels of NK cell-mediated cytotoxicity against SNU-423 compared to HepG2 (**Figure 2B**). Notably these levels were 7-fold and 23-fold higher compared to IL-2 stimulated NK cells for both SNU-423 and HepG2, respectively. OSCSCs, serving as a poorly-differentiated tumor comparison, were lysed at higher levels by untreated and IL-2 stimulated primary NK cells in comparison to OSCCs. And similarly, sNK cells conveyed a higher NK cell-mediated cytotoxicity against both OSCSCs and OSCCs in comparison to primary untreated or IL-2 activated NK cells (**Figure 2C**). **Supplementary Figure 1** shows NK effector cell cytotoxicity against poorly- and well-differentiated HCCs at different E:T ratios.

### IFN-γ mediated modulation of MHC-class I and CD54 on HCCs

**Figure 3:**
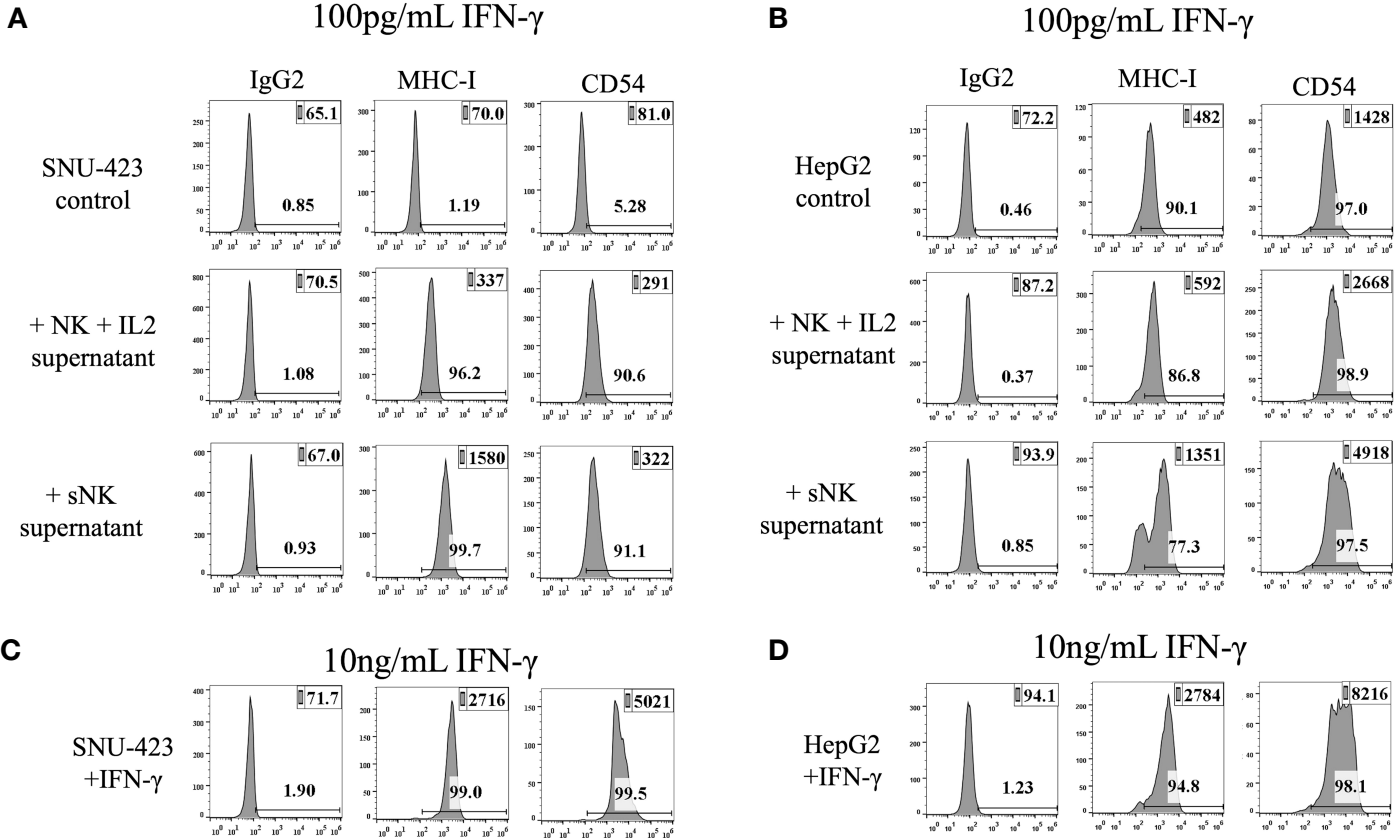
IFN-γ from peripheral IL-2 stimulated NK and supercharged NK cells are able to induce differentiation of HCC cells. **(A)** The addition of IFN-γ to SNU-423 showed an increase in MHC-I and CD54 expression, markers that are seen in more well-differentiated tumors. This trend was similar to what was seen with IFN-γ from sNK, demonstrating the capability of both IL-2 stimulated NK and sNK to cause differentiation of tumors. Differentiation of tumors via upregulation of MHC-I and CD54 can potentially help them become more recognizable by the adaptive immune system and TCR-mediated activity. **(B)** The addition of IFN-γ from various types of NK cells to HepG2 caused a slightly lower expression of MHC-I that was not significant, and no difference in CD54 expression. **(C)** Incubation of SNU-423 at a 100x fold increase in concentration using rhIFN-γ demonstrates upregulation of MHC-I and CD54, but these elevations were not significantly different compared to what was seen with IFN-γ from IL-2 and sNK. **(D)** Incubation of HepG2 in similar conditions showed slightly higher expression of MHC-I but otherwise no significant change in CD-54 expression.

CSCs/poorly differentiated tumors have been shown to undergo further differentiation via NK cell-secreted IFN-γ or rh-IFN-γ (24, 25). To extrapolate this trend to HCCs, SNU-423 and HepG2 were treated with primary IL-2 stimulated NK cell- and sNK cell- derived supernatant containing 100 pg/mL IFN-γ. The addition of IL-2 stimulated primary NK cells and sNK cells derived supernatants was able to consistently upregulate MHC-class I and CD54 in poorly-differentiated HCC (SNU-423), confirming decreased stem-like profile and likely increased susceptibility to TCR-based recognition (**Figure 3A**). Increased surface expression of MHC-class I and CD54 was also seen when well-differentiated HCC (HepG2) were treated with IL-2 stimulated primary NK cells and sNK cells derived supernatants (**Figure 3B**). Incubation of the SNU-423 at a 100x fold increase in IFN-γ concentration at 10 ng/mL of rhIFN-γ showed upregulation of MHC-class I and CD54, similar to what was previously seen with IL-2 stimulated NK and sNK cells derived supernatants (**Figure 3C**). Incubation of HepG2 with 10 ng/mL of resulted in increased surface expression levels of MHC-class I and CD54 on HepG2 (**Figure 3D**).

### Temporal NK effector cell cytotoxicity profile recapitulated in eSight study

**Figure 4:**
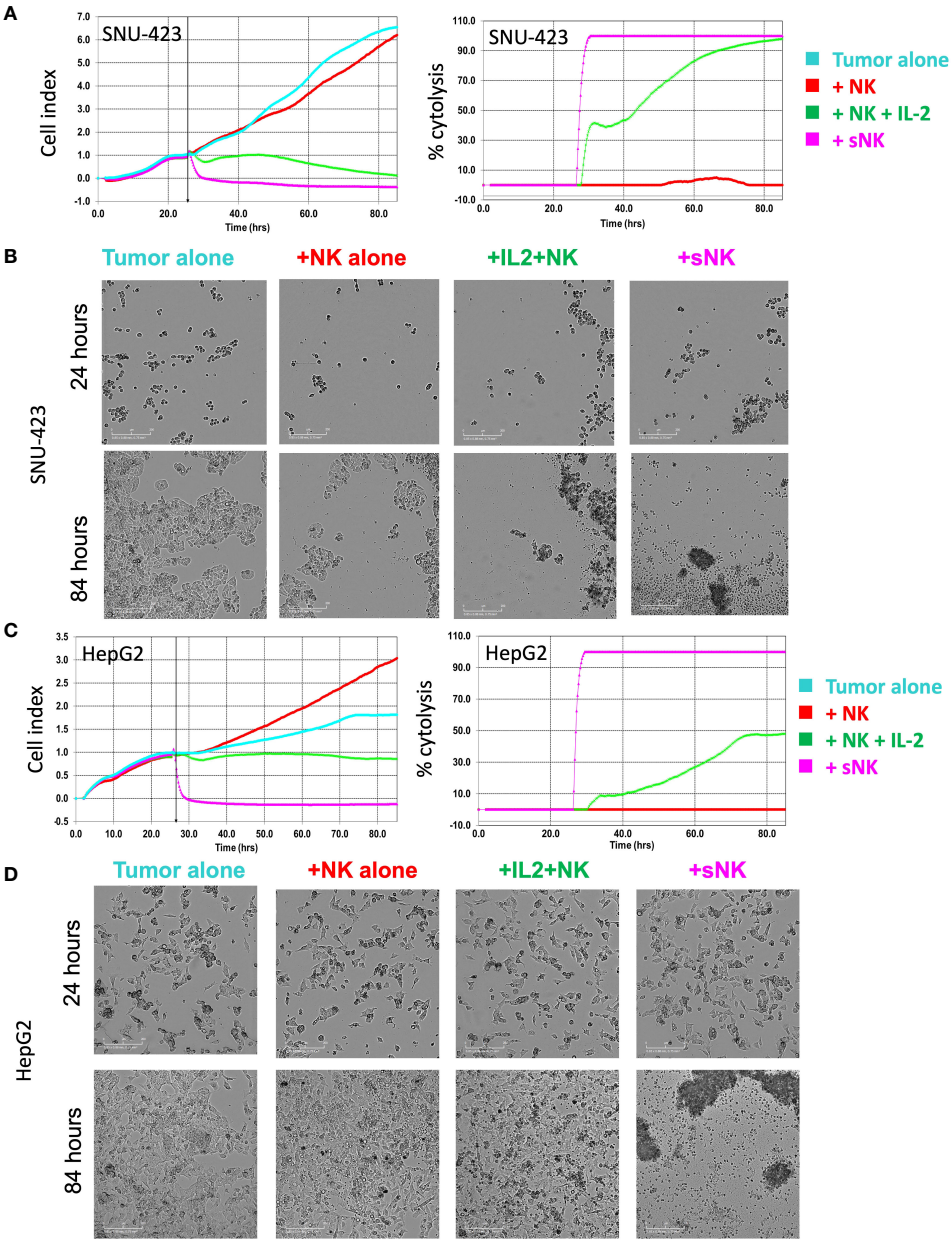
Tracking temporal activity of NK effector cell cytotoxicity. **(A)** Quantitative analysis of cell growth using real-time imaging assay (xCELLigence RTCA eSight platform) compares cell growth with each type of NK cell treatment. Cells were plated for 24 hours and then exposed to NK cell treatments for 60 additional hours (total of 84 hours). (Left) Cell index of SNU-423 tumor control (cyan) and pNK cells (red) were not significantly different from each other at 24 and 48 hours (3.13 ± 0.55 vs 2.77 ±0.18; p = 0.099 and 5.85 ±1.44 vs 4.96 ±0.50; p= 0.287, respectively). IL-2 stimulated NK cells (green) and supercharged NK (sNK) (pink) cells demonstrated lower cell index compared to SNU-423 control and pNK cells across the length of the entire experiment. IL-2 stimulated NK cells had a cell index of 0.98 ± 0.42 and 0.32 ± 0.32 at 24 and 48 hours, respectively. sNK-treated SNU-423 had significantly lower cell index compared to IL-2 stimulated NK cells throughout the study period (-0.24 ±0.19 vs 0.98 ±0.42; p=0.002) at 24 hours, 48 (-0.35 ±0.09 vs 0.32 ±0.32; p=0.007) and 60 hours (-0.37 ±0.09 vs 0.12 ±0.18; p=0.0028), consistent with higher activity level and cytotoxicity. (Right) Percent cytolysis quantifies amount of cell death. sNK cells had a faster and more complete level of SNU-423 death over the course of the experiment. **(B)** Microscopy demonstrating SNU-423 cell death in IL-2 stimulated NK and sNK cells at the end of the study (84 hours). **(C)** (Left) Cell index graph illustrating limited activity of IL-2 stimulated NK cells against HepG2. In contrast, sNK cells retained strong activity against HepG2, causing a significantly lower cell index compared to IL-2 stimulated NK cells at 24 and 48 hours (0.94 ± 0.09 vs 1.26 ±0.07; p = 0.001 and 0.88 ± 0.09 vs 1.79 ± 0.33; p= 0.002, respectively). (Right) sNK reached near complete cytolysis immediately after incubation in HepG2 compared with IL-2 stimulated NK cells. **(D)** Microscopy demonstrating HepG2 cell death in IL-2 stimulated NK and sNK cells compared to control tumor and peripheral NK cells at the end of the study.

Quantitative analysis of both imaging and impedance of each cell line after incubation with NK effector cells are shown in **Figure 4**. After plating the HCC cells at the bottom of the E-Plate for 24 hours, primary untreated NK cells, IL-2 activated primary NK cells, and sNK cells were added to the HCC cells and monitored for an additional 60 hours, for a total of 84 hours (**Figure 4A**). Cell index of the SNU-423 control and tumor with primary untreated NK cells were not significantly different from each other at 24 and 48 hours after plating. Both primary IL-2 stimulated NK cells and sNK cells had significantly lower cell index compared to tumor alone and tumor with primary untreated NK cells at 24 and 48 hours. Qualitatively, after cell plating there was a steady decrease in cell index at 24 and 48 hours with the primary IL-2 stimulated NK cells compared to baseline. sNK cells showed a similar trend in activity but with faster cytotoxic action compared to primary IL-2 stimulated NK cells. sNK cells also showed significantly higher rate of cell death compared to primary IL-2 stimulated NK cells (**Figure 4B**).

**Figure 5:**
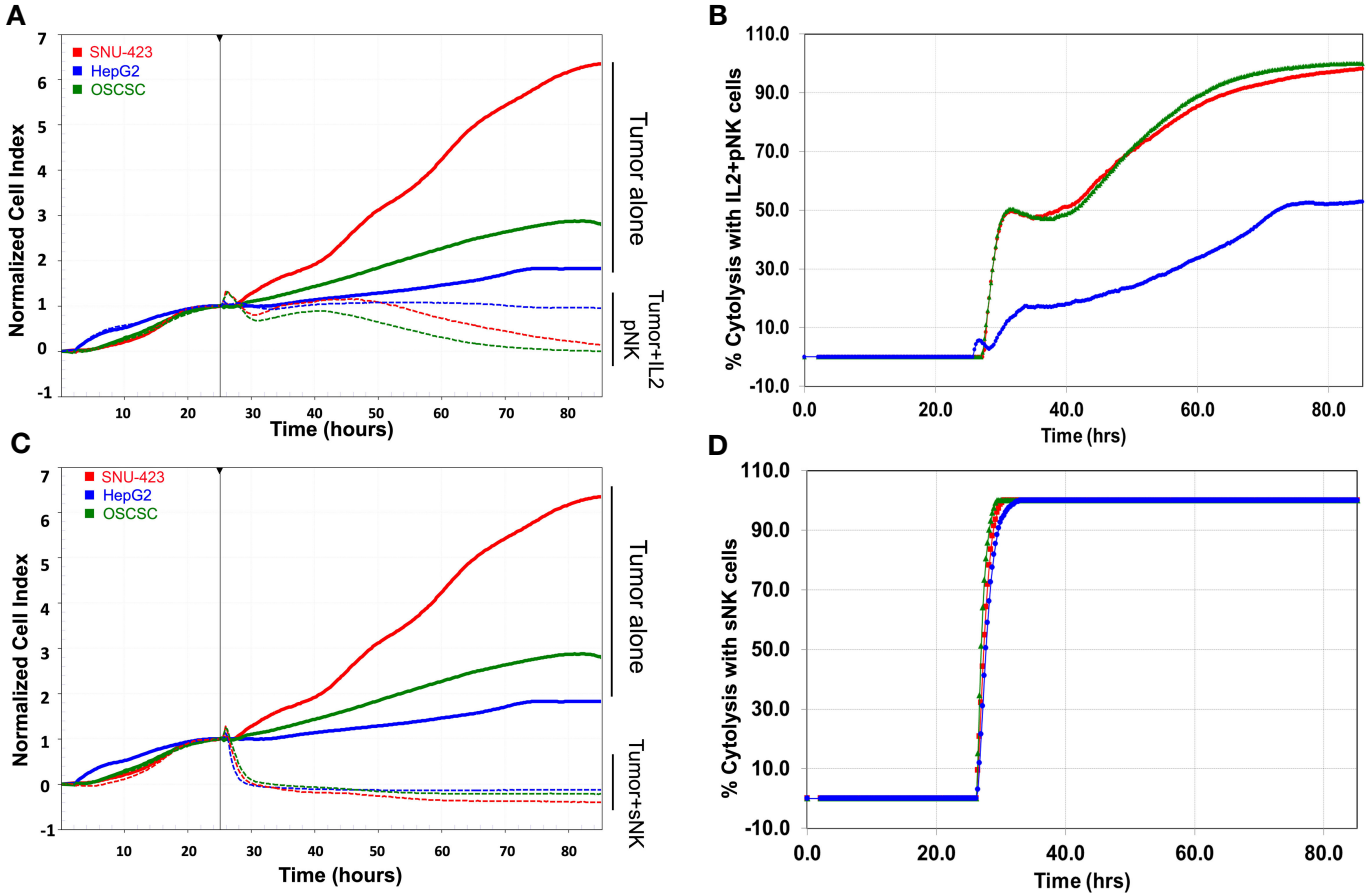
Side by side comparison of targeted tumors with **(A)** IL-2 stimulated primary NK cells and their respective **(B)** percent cytolysis between the poorly differentiated HCC (red), well-differentiated HCC (blue) and OSCSC (poorly differentiated stem-like cancer control). IL-2 stimulated NK cells demonstrated increasing levels of activity (decreasing cell index and increased percent cytolysis) in the poorly differentiated cells (SNU-423 and OSCSC), compared to the well-differentiated HCC (HepG2). **(C)** sNK cells have a quicker response compared to IL-2 stimulated NK across all levels of differentiation (SNU-423, HepG2, and OSCSC) in terms of both normalized cell index and **(D)** percent cytolysis. Note the more rapid decrease in cell index and near instantaneous increase in percent cytolysis in the sNK group compared to the IL-2 stimulated primary NK cells. Unlike IL-stimulated primary NK cells, sNK cells were able to also demonstrate strong activity against well-differentiated HCC (HepG2), at a rate similar to the poorly-differentiated HCC.

With HepG2, there was a comparable trend, albeit to a lower degree compared to SNU-423 (**Figure 4C**). Primary IL-2 stimulated NK cells showed significantly increased activity, evidenced by lower cell index at 24 and 48 hours compared to primary untreated NK exposed cells, although to a lesser degree compared to SNU-423. This was especially pronounced at the later time points of the experiment, where the cell index was significantly less at 60 hours in the sNK cells compared to the primary IL-2 stimulated NK cells (**Figure 4D** **).** Direct comparisons between primary IL-2 stimulated NK cells and sNK cell treatments in SNU-423 and HepG2 are shown in **Figure 5**. Primary IL-2 stimulated NK cells showed more activity against SNU-423 compared to HepG2, with similar cytotoxicity compared to stem-like OSCSC. sNK cells demonstrated significantly faster and more activity against both types of HCC cell lines.

## Discussion

The therapeutic role of NK cells in HCC therapy is an active area of investigation due to its direct modulation by the immunosuppressive nature of the tumor microenvironment. NK cells in HCC patient are known to be defective and have low capacity to target poorly differentiated tumor cells (16). Prior studies have established that activated primary NK cells are able to selectively target pancreatic tumors based on morphological and histopathological staging, and effectively lyse CSCs/poorly differentiated pancreatic tumors (33). The extent of NK cell-mediated killing has been found to be lower in moderately differentiated tumors and only minimally in well-differentiated tumors (22, 32). The selective lysis of pancreatic tumors by NK cells was strongly associated with higher expression of CD44, and no or low expression of CD54 and MHC-class I on CSCs/poorly differentiated histopathology. Conversely, lower expression of CD44 and higher expressions of CD54 and MHC-class I were mainly seen in well-differentiated tumors, leading to lower levels of NK cell cytotoxicity (22, 32). NK cells have also been shown to also target other well-known stem cells, including human mesenchymal stem cells, dental pulp stem cells, embryonic stem cells and induced pluripotent stem cells (15, 29, 34). While heterogeneity is expected within CSCs, tumors classified as CSCs/poorly differentiated tumors in general have been noted to have lower MHC-class I expression and the higher levels of recognition and subsequent lysis by the primary activated NK cells compared to well-differentiated tumors (15).

In this study, we investigated the stage of histopathologic stage of differentiation of HCCs and correlated it with its level of NK cell-mediated cytotoxicity. We showed that *in-vitro* HCCs on opposite ends of histopathologic differentiation also exhibited opposite levels of stem-like cell markers respectively. CSCs/poorly-differentiated HCCs (SNU-423) recapitulated the surface marker profile seen in other stem-like solid tumors, namely low expression of MHC-class I and CD54, and high expression of CD44, and they were subsequently found to be susceptible to primary activated NK cells. HepG2 cells, which exhibited the surface marker phenotype of well-differentiated tumors, had correspondingly much less lysis by primary activated NK cells.

Due to the defective nature of NK cells in cancer patients, there is an unmet need for a treatment strategy that can eliminate the CSCs/poorly differentiated HCCs. To that end, we have developed a strategy to augment the survival and function of NK cells in tumor microenvironment using sNK cells. Traditionally, NK cells target CSCs via two pathways 1) direct NK-cell mediated killing, and 2) induced target tumor differentiation through secreted IFN-γ and TNF-α (24). sNK cells amplify both of these pathways by exhibiting significantly higher levels of cytotoxicity and cytokine secretion in comparison to primary activated NK cells, and this behavior was confirmed in this study against the poorly-differentiated HCC (21). The precise mechanisms for the higher levels of cytotoxicity in sNK cells compared to IL-2 stimulated NK cells remain an area of active investigation (21, 22, 24, 32). Briefly, granule release, particularly granzyme B, has been noted to increase in sNK cells, as well as their regulators such as cystatin F and Cathepsin C (manuscript in prep) (32). Thus, short-term sNK cell cytotoxicity is likely mediated through granzyme B and potentially through other granules in sNK cells, while long-term surveillance and destruction likely relates to increased sNK expressing death receptors such as TNF-α, Fas-L and Trail binding to death receptors on tumor cells. The second mechanism of action of sNK cells relating to tumor differentiation is based on the fact that poorly-differentiated HCCs with low MHC-I expression have theoretically been able to escape MHC-based antigen presentation. However, with respect to sNK-based cytokine secretion of IFN-γ, there is a potential pathway to now upregulate MHC-I in the poorly-differentiated HCC, leading to MHC-based antigen presentation and recognition and subsequent T cell activation (35, 36). Altogether, these findings suggest that NK-cell based immunotherapy can leverage this two-pronged approach to effectively treat poorly-differentiated HCC. Another unique attribute of sNK cells noted in this study was their ability to also lyse well-differentiated HCCs with high MHC-class I expression. This feature makes sNK cells an exquisitely potent therapeutic modality because they not only target CSCs/poorly differentiated tumors, but can also lyse tumors that participate in the MHC-class I antigen presentation pathway, a function historically associated with CD8+ T cytotoxicity. Therefore, this study suggests that sNK cells have the augmented cytotoxicity profile of traditional NK cells, and can also carry the functions of CD8+ T cells. Future investigations of cell-based therapies should focus on characterizing such immune cells that can simultaneously leverage both innate and adaptive cytotoxicity pathways.

There are many key advantages to using NK cells for HCC immunotherapy. NK cells do not require specific antigen sensitization expressed on an HLA allotype. This minimizes the risk for tumor antigen mutations or tumor escape variants that lead to immunotherapy resistance. Secondly, NK cells are readily isolated and expanded *ex-vivo*, allowing for use in adoptive or allogeneic cell therapies. Lastly, NK cells have comparatively shorter lifespan compared to clonally-expanded T cells, which decreases the risk for off-target effects. Along with these advantages, however, multiple challenges remain with using NK cell therapy. While the short lifespan is desirable to with regards to minimizing side effects, NK cell therapies still need to persist long enough for a therapeutic effect. IL-2 stimulated NK cells have increased levels of function but are difficult to expand to the levels needed for cellular therapy. The expansion process is highly dependent on *in-vivo* cytokine support, which may be difficult to maintain in a systemic environment. However, this may be facilitated with catheter-directed techniques that are already being used in HCC patients. NK cells in HCC patients are also known to be defective compared to healthy controls, meaning that existing *ex-vivo* expansion methods may not be adequate. Allogeneic supercharged NK cells may provide a potential solution. Lastly, NK cells may also have difficulty in surviving and penetrating the HCC tumor microenvironment. This challenge may also be overcome through the use of sNK cells since it has been shown that they have increased survival, penetration and lysis of glioblastoma tumors, in addition to image-guided needle or transcatheter delivery directly into the tumor microenvironment (37).

NK cells have shown great potential in eliminating target cells in standard *in-vitro* cytotoxicity assays. However, their efficacy in three-dimensional tumor models have been shown to be reduced due to a 6-fold decrease in motility (38). Interventional radiology techniques using intra-arterial catheter-directed approaches offers a potential solution to improve localization. Early stage HCCs are already being treated using image-guided techniques such as transarterial chemoembolization or radioembolization. Leveraging these techniques for NK cell therapies allows for direct injection of cytokine support and direct targeting of the tumor via hypervascular anatomy associated with HCCs. Thermal ablation, either using radiofrequency or microwave energy also offers a promising heat-based synergy with NK cell therapy. Given the limited success of monotherapy with systemic primary NK cell therapy, it is likely that future studies targeting HCC will require combination or neoadjuvant therapies to maximize its potential.

There are some limitations of this study that need to be taken into consideration. First, while the HepG2 cell line has historically been utilized as a well-differentiated HCC cell line, there are controversies with its origin being potentially from a hepatoblastoma (39). However, given that its histopathologic phenotype retains most of the metabolic and immunologic functions of normal hepatocytes and has similar mutations to HCC, the HepG2 model remains a suitable model for a well-differentiated HCC. The second limitation is that we only evaluated a single cell line of each type of differentiation. While there are other cell lines available, most are also poorly-differentiated HCCs. Thus, these two lines were selected to represent the opposite sides of the differentiation spectrum. Successful clinical translation will also require additional *in-vitro* studies specifically looking at mechanism of cytotoxicity between sNK cells and HCCs. For example, quantification of interactions of NK cell receptors and ligands can facilitate effective design of targeted NK cell-based therapies, especially if sNK cells will be combined with thermal ablation or transarterial liver directed therapies. Finally, these results need to be confirmed in an *in-vivo* model with a true HCC tumor microenvironment to confirm its efficacy.

In conclusion, poorly-differentiated HCC cell lines recapitulated the stem-like profile seen in other poorly-differentiated solid tumors. Poorly-differentiated HCCs were more susceptible to activated primary NK cell- and supercharged NK cell-based therapies. The success of *in-vitro* NK cell therapy against HCC can be translated in an *in-vivo* model, potentially using targeted needle-based or catheter-based delivery that are already part of the HCC treatment armamentarium.

## Data availability statement

The raw data supporting the conclusions of this article will be made available by the authors, without undue reservation.

## Ethics statement

The studies involving humans were approved by UCLA Institutional Review Board (IRB#11-000781). The studies were conducted in accordance with the local legislation and institutional requirements. The participants provided their written informed consent to participate in this study.

## Author contributions

JC: Conceptualization, Data curation, Formal Analysis, Funding acquisition, Investigation, Methodology, Project administration, Supervision, Validation, Visualization, Writing – original draft, Writing – review & editing. P-CC: Data curation, Formal Analysis, Methodology, Project administration, Validation, Writing – original draft, Writing – review & editing. JP: Data curation, Methodology, Project administration, Writing – original draft, Writing – review & editing. C-QN: Data curation, Formal Analysis, Investigation, Methodology, Writing – review & editing. KK: Writing – original draft, Writing – review & editing. SR: Writing – review & editing. AJ: Formal Analysis, Funding acquisition, Project administration, Writing – original draft, Writing – review & editing.
