# Supplementary material for: Characterizing hepatocellular carcinoma stem markers and their corresponding susceptibility to NK-cell based immunotherapy
Source: Front Immunol. 2023 Oct 26;14:1284669. doi: 10.3389/fimmu.2023.1284669 (PMC10637628; doi:10.3389/fimmu.2023.1284669)

**Supplementary Figure 1**

A 2-fold serial dilution was initiated starting from 5 x 10^4^ primary untreated NK, IL-2 treated NK, and sNK cells. Subsequently, 1 x 10^4^ of ^51^Cr-labeled HCC tumor cells were added and co-cultured for a period of four hours to attain an initial E:T ratio of 5 to 1. A) Primary untreated NK cells exhibited a higher levels of cytotoxicity against poorly-differentiated HCCs (SNU-423) compared to well-differentiated HCCs (HepG2). This trend was also seen in B) IL-2 stimulated NK cells, and C) sNK cells. This pattern was also seen in the comparison in the control poorly differentiated OSCSCs and well-differentiated OSCC with D) primary NK cells, B) IL-2 stimulated NK cells and C) sNK cells.


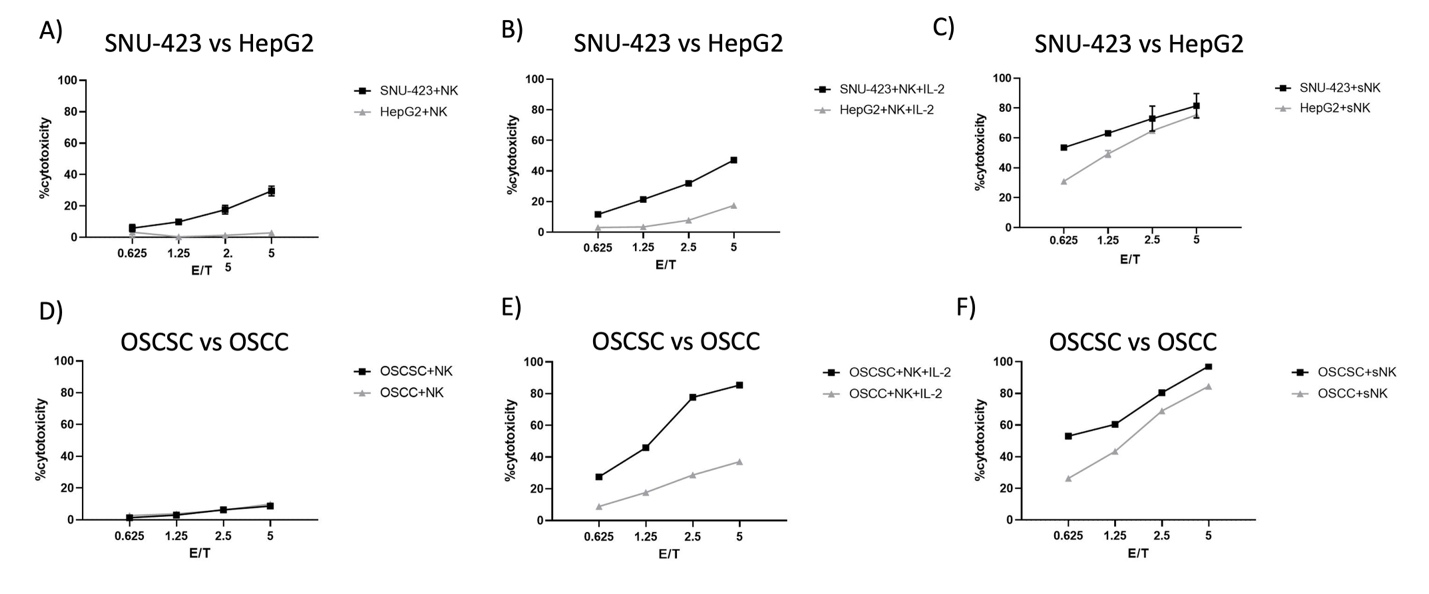

Supplement: Supplementary file 1 [file DataSheet_1.docx]
